# Uncovering the effectiveness of anthropomorphic communication on the country-of-origin stereotypes from the perspective of psychological elements

**DOI:** 10.1038/s41598-023-45963-x

**Published:** 2023-10-31

**Authors:** Shizhen Bai, Wei Zhang, Lingyun Chu

**Affiliations:** School of Management, Harbin University of Commerce, Harbin, 150028 People’s Republic of China

**Keywords:** Psychology, Human behaviour

## Abstract

With the development of social media, interactive activities such as anthropomorphic communications are more accessible and popular. The country-of-origin(COO) stereotype is one of the most important factors which influences individuals' attitudes toward brands. This study aims to investigate the feasibility and validity of anthropomorphic communication via social media on COO stereotypes in international competitions. Experimental results indicate that the strategy of using anthropomorphic communication has positive effects for developing countries, but not the same for developed countries; the perceived social presence mediates the relationship between anthropomorphic communication and COO stereotypes of developing countries. However, the mediating effect is not obvious in developed countries, the influence of anthropomorphic communication is effective and feasible only when individuals’ mindset is global. The theoretical model in this paper is a useful supplement to the existing achievements of anthropomorphism and COO stereotypes, it provides a reference for enterprises in developing countries to use anthropomorphic strategies accurately to reduce negative COO stereotypes and improve international competitiveness, it also gives suggestions for companies in developed countries to adopt anthropomorphic communication strategies cautiously.

## Introduction

Anthropomorphism can increase individuals’ potential “relevance” to the brand, and maintain long-term emotional relationships between brands and customers, which could improve brand attachment significantly^1^. Anthropomorphism also excites individuals’ shopping happiness and improves their loyalty to brands^2^. More and more enterprises began to adopt anthropomorphic strategies via social media to communicate with customers, such as the “Wangzai Club”, which is a social media account of a food enterprise, and the “Haier brothers”, a brand account which has opened official webpages on Facebook, they usually share interesting stories with users about their daily life and disseminate advertisements in a human being tone, carrying out interaction activities with individuals, which have effectively established positive brand images in the environment of international competition. The interactive activities between brands and individuals evoke vividness and sociability, which induces a heightened sense of social presence^3^. Weidlich argued that social presence is an important construct to understand psychosocial processing^4^; Fan investigated the influence of social presence on brand trust^5^; Sun discovered the relationship between social presence and user-generated content by quantitative methods^6^. Anthropomorphic elements elicit aesthetic appeal, and the sense of social presence reduces the distance between individuals and brands^3,7^, these make people feel that they are in an interactive space and draws their attentions to the brand, could the feelings further decrease individuals’ country-of-origin stereotypes? Limited research has been conducted on the subject, so this paper tries to fill this gap.

Individuals often associate product evaluations with their country-of-origin image. They generally prefer products which are made in developed countries and propose them with fewer risks or better qualities^8^. For some developing countries, COO information may be a disadvantage for their brands. Recently, studies have found that brand image also has an impact on the country’s image^9^, and the interaction of the brand image and the country's image may lead to the formation of deeper stereotypes, so how to reduce COO stereotypes becomes more and more important for developing countries in international competition. Previous studies and real-world examples have shown the advantages of anthropomorphism, when a brand adopts human-like language in communication, it may excite individuals’ positive attitudes toward the producrt^10^, the latest research findings reveal the effects of anthropomorphism on customers’ satisfaction^11^ and product recommendations^12^. Psychological factors such as individuals’ mindset could also influence their preference for products. Septianto showed that individuals with fixed (vs. growth) mindset would lead to higher preferences for typical products^13^; Rubin examined the effect of individuals’ abstract mindset vs. concrete mindset on purchase intention^14^; Salnikova explored how individuals’ global mindset vs. local mindset affect brand engagement^15^. We expect to further the research on COO stereotypes and explore psychological factors such as the perceived social presence and individuals’ mindset which influences the effectiveness of anthropomorphism on COO stereotypes, to deepen the research field of anthropomorphism and stereotypes. This paper adopts the method of empirical studies to analyze these relevant variables. Based on reviewing literatures related to influencing factors of COO effects^16^, and the effectiveness of anthropomorphism^10,17^, it could fill in at least three research gaps:

First, the COO stereotype influences individuals’ attitudes toward brands, it has an important influence on purchasing behaviors in our daily life. Anthropomorphic communication via social media elicits emotional reactions to individuals, however, current literature on anthropomorphic communication mainly focuses on relationships between individuals and brands, such as anthropomorphic communication reduces the distance between customers and brands^10^, anthropomorphism leads to individuals’ brand trust^17^, or what does anthropomorphic communication do to individuals’ brand awareness^18^. In fact, it is little but essential to consider the anthropomorphic influence on COO stereotypes; Second, we try to analyze the interactive communication effects by psychological experiments and introduce the variable of social presence to the COO research area, further enriching the influence of individuals’ psychological variation triggered by anthropomorphic communication on COO stereotypes, extending the application scenario of social presence theory and the theory of stereotype; Additionally, information perception may excite different mindset processing, we intend to explore how individuals’ mindset affects the validity of anthropomorphism on COO stereotypes in the perspective of cognitive psychology, and provides a useful reference for the implementation of anthropomorphic strategy in international marketing.

When we use the same anthropomorphic communication for brand promotion in different countries (developed or developing countries), the practical effect of the anthropomorphic communication may be different, some factors such as psychological or media environmental elements may also influence the results. Therefore, we mainly focus on studying these three questions to make our study more rigorous and in-depth:

**Question 1:** Could anthropomorphic communication reduce negative COO stereotypes of developing countries? What about for developed countries?

**Question 2:** Are individuals' mindset and perceived social presence important factors that affect the effectiveness of anthropomorphism on COO stereotypes? What is the internal mechanism between them?

**Question 3:** How to make advertisement and brand international promotion strategies more effective by using anthropomorphic communication strategies more precisely in the social media environment?

The research structure is as follows: in the literature review, the usefulness of anthropomorphic communication, influenced factors of COO stereotypes, such as the social presence, mindset processing, and related theories are collated and analyzed; Then research hypotheses are proposed in the hypotheses development section; The method part contains the experimental design and subject selection, data recovery and data processing; Conclusions, theoretical and practical implications, limitations and future research are organized in the conclusion and discussion part.

## Literature review and hypotheses deduction

### Theoretical background

*Schema theory.* Schema theory is important for human cognition. Based on schema theory, people can filter, screen, and sort out external stimuli, make information into a systematic cognition, and establish a new schema. Humans are more familiar with objects which imitate themselves, schema theory helps individuals form a knowledge system and makes them understand objects better^19^. Anthropomorphic communication, just like human communicates with others, it stimulates human schema and makes human beings better understand what the advertisement introduced, and better familiarize themselves with product information; Schema theory is also related to COO stereotypes. Individuals put country-associated information into a schema^9^, the schema provides individuals a frame for forming brand evaluations, which interpret new information as an anchor^20^. Previous studies found that individuals may associate country images with their personality traits as part of the mental schema, when brand information is insufficient, people are more likely to give evaluations based on their schema that has been formed before^21^. COO stereotype associations can be positive, negative, or neither. All of them constitute cognitive schema for individuals, and individuals form different cognitive schemas for brands based on their memories and impression formation of countries where brands originate. Once the beliefs and attitudes towards different countries are formed, it would affect individuals' brand attitudes under psychological concerns.

Based on the human schema theory, we find that people pay more attention to products with human characteristics, and it inspires more positive attitudes toward brands for would-be-purchasers^10^. The human schema also evokes COO information in individuals’ minds, which makes people associate the brand with their national image or products which the country produced in the past. Schema theory is closely related to both anthropomorphism and COO stereotypes. Based on the main clue of schema theory, this paper will further study the impact of anthropomorphic communication on COO stereotypes.

### The effect of anthropomorphic communication on country-of-origin stereotypes

From the perspective of marketing, COO image refers to individuals’ overall perception of products’ quality which is made in different countries^22^. COO stereotypes can influence brand evaluations and purchase intentions significantly^16^. When individuals are unfamiliar with the brand, their prior cognitive schema knowledge becomes important for brand evaluations. When the perception of brands is mostly based on individuals’ prior knowledge, COO stereotypes are formed. People believe that products made in developed countries have better qualities^8^, which leads to brand cognitive bias for different countries, especially for developing countries. The negative COO stereotype is harmful to global marketing. Magnusson provided a reference on how COO information affected individuals’ brand attitudes^23^. Mbs believed that COO stereotypes were valuable resources and this effect could be transformed into national advantage^24^. Other external cues such as information effectiveness^25^, implicit personality^26^ also play modify roles on COO stereotypes. COO stereotypes appear relatively weak or insignificant when some other external cues are available.

Anthropomorphic communication via social media encourages people to perceive the brand as a real person, he/she chats with individuals about unrelated subjects, using language such as "we, let's, little sister, I, etc." The character features, such as a smiling face, character action, etc. are attached to the brand publicity poster, which make individuals feel that the brand is alive. Anthropomorphic communication helps individuals understand brands more deeply when they face complex situations^27^, Gretry proposed that using anthropomorphic communication could improve individuals’ positive attitudes toward brands^10^, Maroufkhani identified the mechanism of interactive voice assistants toward brands’ loyalty^28^. Anthropomorphic communication brings human emotions to brands, delivers brand-relevant information to individuals, and satisfies individuals’ basic social interaction needs in the process of communication and marketing. It generates the pleasure of interpersonal communication that only exists in the real human world, the irrelevant information and anthropomorphic tone may reduce advertising pressure and trigger people’s social interaction more successfully in the content of social media, it elicits emotional reactions from individuals, makes them understand brands better, and then reduce COO stereotypes of the brand. Above this, we propose that:

H1: Anthropomorphic communication via social media reduces country-of-origin stereotypes.

### The mediating effect of social presence

The sense of presence is the sensory experience of individuals, it contains spatial presence and social presence^29^. Spatial presence is the degree to which individuals feel their real existence in the virtual environment, while social presence emphasizes feelings of being together with others, it is the degree to which individuals interact and connect with others via social media^3^. For this research, we mainly focus on the importance of anthropomorphic communication in the content of social media, which belongs to the concept of social presence. Kreijins found that the sense of social presence affects the result of social interaction, which is an important concept in network group communication^30^. Social presence makes individuals feel sociable, warm, sensitive, and active. Vividness and interactivity are the main components of the communication system. It generates immersive feelings and improves individuals’ attitudes toward brands. Herrando found the mediated effect of social presence on attitudes and social commerce WOM^31^; Van Brakel showed that social presence was positively associated with users' subjective well-being^32^. Hossain found social presence had a significant connection to social sharing and shopping intention^33^. However, relevant literature research about the impact of social presence on COO stereotypes is still limited.

In the network environment, enterprises and individuals lack face-to-face communications, some intimidating and tedious information released by the brand may decrease the consolation generated in interpersonal communications. We propose that anthropomorphic communication plays an important role in social media scenes, it provides individuals with kinds of feelings that originally existed in the real world, triggers human schema and anthropomorphic perceptions, and also a sense of social presence. The sense of presence enhances individuals’ brand recognition^32^, improves their satisfaction, and increases consumer behavioral intention^3^. Through the influence of social presence, the inner self of individuals may be aroused, they will meet their own needs from the perspective of conformity psychology, compensation psychology, emotional satisfaction, and self-realization, which enhance individuals' satisfaction and happiness in the purchasing process, and then reduce the COO stereotypes about the brands or products. Accordingly, we put forward our hypothesis:

H2: Social presence plays a mediating role in anthropomorphic communication and COO stereotypes.

### The moderating role of individuals’ mindset

The mindset is how people pay attention to information and process it. Based on differences in individuals’ information processing patterns, precious researchers have studied the fixed vs. growth mindset^13^, the abstract vs concrete mindset^14^, and the global vs. local mindset^34^, etc. Aljukhadar and Senecal found the effectiveness of involvement in the recommender system was contingent on individuals’ mindset^35^. Bakis and Kitapci examined the moderating role of individuals’ mindset in the relationship between symbolic attributes and the purchase intention of green products^36^. Researchers examined the different influences of mindset (abstract vs. concrete) or (global vs. local) on purchase intention^14,37^. Gao claimed that when individuals' local identity was inspired, they were less likely to be price sensitive^38^. Liu discovered individuals with a global mindset might find a similarity between the stimulus and corporate social responsibility^39^. We introduce the global vs. local mindset processing role into the field of anthropomorphism, to find the interaction effects of anthropomorphic communication and mindset processing on COO stereotypes.

Individuals with global mindset are more likely to see the outside world as a whole, they pay more attention to the similarity of objects^40^. When individuals are stimulated with global mindset, they may integrate information with their prior knowledge and activate information that already exists in their memory. Based on this cognitive structure, people make their own decisions on these new stimuli according to their existing knowledge domain, and make their evaluations according to the previous impressions. However, individuals with local mindset tend to perceive details from the external world, they focus more on the differences between objects and create a contrast effect. In the condition of global mindset, individuals’ evaluations of new things are associated with their prior cognition knowledge domain, which may create the similar stereotype as before, anthropomorphic communication could reduce the COO stereotype through interactive activities; In the local state of mindset, people perceive different details in narrow categories, and pay more attention to the object itself, anthropomorphic communication strategy may not be effective when advertisements stimulating individuals’ local mindset. So we propose that:

H3: Individuals’ mindset (local vs. global) moderates the relationship between anthropomorphic communication and COO stereotypes.

## Methodology

The methodology section contains a pretest and three main studies. All experimental protocols were approved by the Star Platform Institution of China, and all studies were carried out in accordance with relevant guidelines and regulations. Informed consents were obtained from all subjects and/or their legal guardian(s). To better understand the impact of different stimuli on the variations of consumer psychology and the country-of-origin stereotypes, we take three main experiments to investigate the impact of different communication modality (anthropomorphism vs. non-anthropomorphism) on COO stereotypes. The mediating mechanisms of social presence and the moderating role of individuals’ mindset is to be further investigated.

### Study 1

Study 1 is to examine the impact of anthropomorphic communication on COO stereotypes in the context of social media (H1).

#### Research design

Participants were told that this activity was an investigation of new brand of juice, and the brand’s relevant information was revealed by social media. People were asked to give evaluations on the product by their true feelings and gave feedbacks from the perspective of an outsider^41^.

Study 1 was a 2 (anthropomorphism vs. non-anthropomorphism) × 3 (developed country vs. developing country vs. control group) between-subjects design. The control group did not display any country-of-origin information. There were 286 valid subjects in the experiment (age range = 18 to 69, M = 32.65,58.7% female). We took a fictitious brand named “Belly” as the target object, and adopted two different styles of communication modality to broadcast the brand information. In the condition of an anthropomorphic communication group, a virtual spokesman via social media chatted with participants and said: “Hi, I’m Belly, I am full of 100% quality pulp, the smooth and mellow taste will move your heart. Take me with you, let's share the wonderful life!” For the non-anthropomorphic condition group, the spokesman said: “This is the juice of Belly brand, it is made up of 100% premium pulp and with silky, creamy taste. It is a good choice to taste it and have it in your life.”

We chose Australia to represent developed countries, and Philippines to represent developing countries. Participants were arranged randomly into one of the six groups: anthropomorphic, Australia (Naa = 48); anthropomorphic, Philippines (Nap = 48); anthropomorphic, control (Nnc = 49), non-anthropomorphic, Australia (Nna = 49); non-anthropomorphic, Philippines (Nnp = 46); non-anthropomorphic, control (Nnc = 46). They got the description which the spokesman released via social media and then answered a series of questions on the list.

Anthropomorphic measurement items included: (1) I feel Belly has come alive; (2)I feel Belly like a person^42^. The measurement of COO stereotypes contains four areas, including product design, workmanship, prestige, and innovativeness^43^. Evaluation answers were measured by using a seven-point scale items (1 = ”low” and 7 = ”high”). Emotional state was measured by 4 questions to rule out the effects of people’s emotions^44^. Finally, participants reported the answers of these items.

#### Results and discussion

*Anthropomorphism.* Results showed that the degree of anthropomorphism in the anthropomorphic group was higher than that in the non-anthropomorphic group significantly (M_an_ = 5.33, SD = 1.07; M_non_ = 4.97, SD = 1.47, t = 2.36, df = 255, p = 0.00, d = 0.28); Differences in the emotional state between the two groups was not obvious (M_an_ = 5.35, SD = 1.00; M_non_ = 5.40, SD = 1.13, t = − 0.42, df = 284, p = 0.68, d = − 0.05), so we concluded that the results weren’t influenced by participants’ sentiment, the anthropomorphic manipulation was successful.

*COO effect.* We conducted a 2 (anthropomorphic vs. non-anthropomorphic) × 3(developed country vs. developing country vs. control group) multivariate analysis of variance, and took product evaluations of the origin country as dependent variable. The interaction effect between communication modality (anthropomorphic vs. non-anthropomorphic) and COO information (developed country vs. developing country vs. control group) was significant (F = 6.10, df = 2, p = 0.00), results were shown in Table 1.

**Table 1:** Multivariate analysis of variance for product evaluations of the origin country.

Source	df	MS	F value	P value	Eta^2^
Communication modality	1	0.45	0.39	0.53	0.00
COO information	2	4.13	3.60	0.03	0.03
Communication modality*COO information	2	6.98	6.10	0.00	0.04
Error	280	1.15			

Simple effect results showed that, in developed countries, the anthropomorphic group reported lower evaluations of the product than the non-anthropomorphic group significantly (M_an_ = 5.22, M_non_ = 5.70, p = 0.01); in developing countries, the anthropomorphic group reported higher evaluations of the product than that in the non-anthropomorphic group significantly (M_an_ = 5.35, M_non_ = 4.75, p = 0.02).

**Figure 1:**
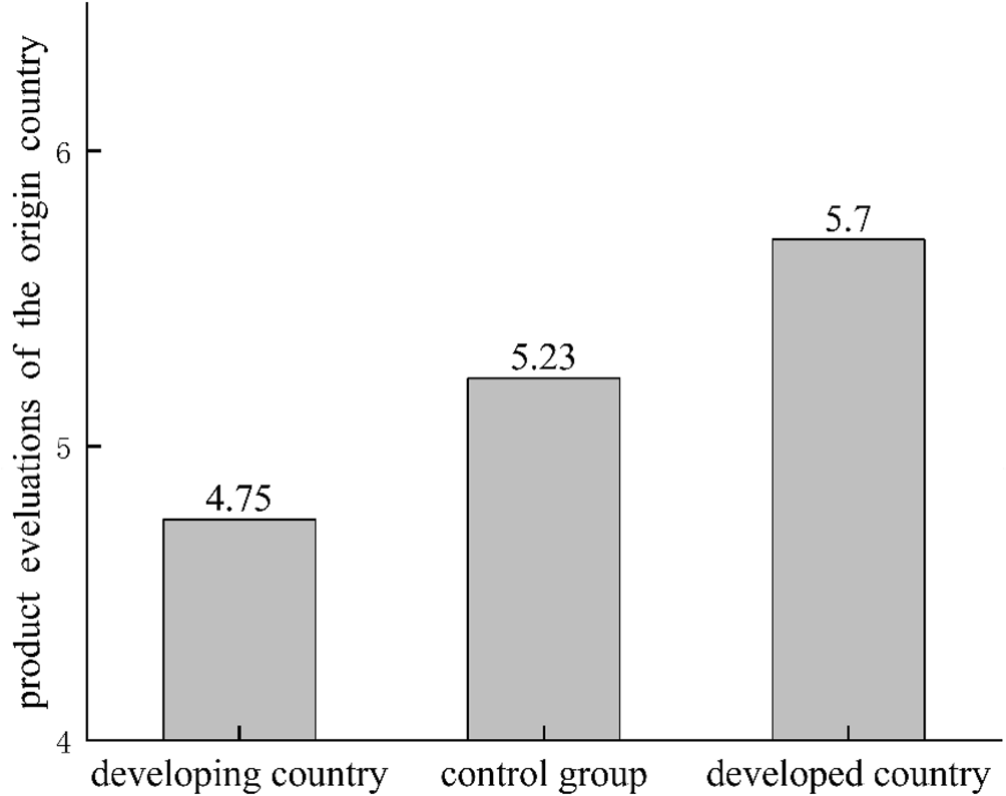
Product evaluations for different country groups in non-anthropomorphic conditions.

**Figure 2:**
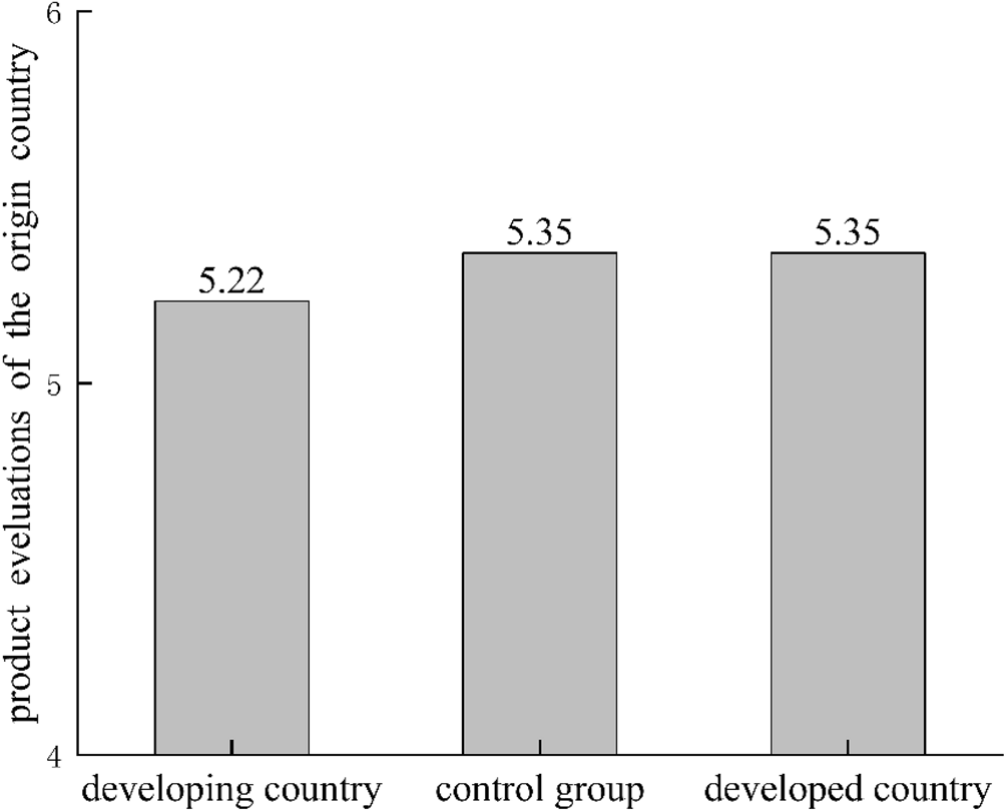
Product evaluations for different country groups in anthropomorphic conditions.

*In the non-anthropomorphic group.* Participants in the developing countries reported lower product evaluations than those in the control group (M_non, ctrl_ = 5.23, SD = 1.01; M_non, phi_ = 4.75, SD = 1.44, t = 1.84, df = 81, p = 0.07, d = 0.38). Participants in the developed country group gave higher evaluations on the target object than the control group (M_non, aus_ = 5.70, SD = 0.96; M_non, ctrl_ = 5.23, SD = 1.01, t = 2.35, df = 93, p = 0.02, d = 0.48). Differences in product evaluations between these three groups were shown in Fig. 1:

*In the anthropomorphic group.* Results showed insignificant difference between the three groups (M_an, ctrl_ = 5.35, SD = 1.06; M_an, aus_ = 5.22, SD = 0.86, t = 0.63, df = 95, p = 0.53, d = 0.13); (M_an, phi_ = 5.35, SD = 1.01; M_an, ctrl_ = 5.35, SD = 1.06, t = 0.01, df = 95, p = 0.99, d = 0.002). Differences between these three groups were not obvious, results were shown in Fig. 2:

Results of study 1 showed that anthropomorphic communication via social media reduced COO stereotypes, which confirmed hypothesis 1. In the social media environment, anthropomorphic communication not only reduced the negative effects of developing countries but also reduced the positive effects of developed countries. The findings further support Feng’s previous research about anthropomorphism and country-of-origin stereotypes^25^. It provides a reference for enterprises to adopt anthropomorphic communication strategies effectively and cautiously.

### Study 2

The purpose of this study is to explore the mechanism of social presence in the relationship between anthropomorphism and COO stereotypes, meanwhile it is aimed to test H2.

#### Pretest

A pretest (N = 73, 74% female) was to test COO stereotypes for developing countries and developed countries. We took France as the representative of developed countries and Vietnam as the representative of developing countries. We chose sports shoes as the target object. Participants were assigned to one of the two groups randomly with different COO information. The advertiser showed participants pictures of sneakers and country-of-origin information (France or Vietnam), and then asked participants to answer 4 questions to test the COO stereotypes^43^. The rating was on a 7-point scale from 1 to 7, where 1 = “low” and 7 = “high”.

After excluding the influencing factors of individuals’ sentiment^44^ and psychological distance^41^, we found that evaluations of the product were different significantly between the two groups, individuals in the developing country group(Vietnam) made lower evaluations of the product than those in the developed country group(France) (M_VN_ = 3.65, SD = 1.77, M_FR_ = 4.80, SD = 1.70, t = 2.83, df = 71, p = 0.01, d = 0.66), indicating that there were COO stereotypes on products between developing countries and developed countries, and the two countries((France vs. Vietnam)made a successful manipulation of COO information to represent developed countries and developing countries respectively.

#### Research design

In study 2, we took a 2 (anthropomorphic vs. non-anthropomorphic) × 3 (developed country vs. developing country vs. control group) between-subjects design. Product information was released by the spokesman via social media just like in study 1, we recruited 190 valid participants (age range = 17–69, 52.1% female) on the internet and they were randomly assigned to each group. We took sports shoes as the target product and named the brand “Welly”. The same as study 1, two types of communication were introduced to release the product information. For the anthropomorphic group, the spokesperson chatted with participants about unrelated items, such as their interests and pleasant experiences. The brand spokesperson was like an old friend of these participants and said: “Hi, I’m Welly, I am designed by top designers, possessing high quality and good after-sales service. Take me home, I’ll share you the comfortable and wonderful life.” For the non-anthropomorphic group, the spokesman said: “The shoes of Welly brand are created by top designers. It is with reliable quality and perfect after-sales service. It will bring you comfortable and wonderful feelings if you buy them home.”

Just like the pretest, France was selected to represent developed countries, Vietnam was taken to represent the developing countries. Participants evaluated the product from the third people’s perspective^41^. They were randomly allocated to one of the six conditions: anthropomorphism, developed country(N_an, Fr_ = 30); anthropomorphism, developing country(N_an, Vi_ = 31); anthropomorphism, control group(N_an, ctrl_ = 35); non-anthropomorphism, developed country(N_non, Fr_ = 32); non-anthropomorphism, developing country(N_non, Vi_ = 31); non-anthropomorphism, control group(N_non,ctrl_ = 31). After getting the information from the advertisement, participants reported their evaluations. Next, they filled up a list which contained a series of questions, such as emotional state^44^, familiarity with the brand, and other question items. Participants reported the degree of anthropomorphism by two items^42^. The degree of social presence was then tested by three items, such as “feel a sense of human contact; feel a sense of sociability; feel a kind of human’s warmth”^45^.

#### Results and discussion

*Anthropomorphism.* We conducted an independent sample T-test for the two groups(anthropomorphic vs. non-anthropomorphic), the anthropomorphic group gave a higher level of anthropomorphism than the non-anthropomorphic group (M_an_ = 6.28, SD = 0.89; M_non_ = 5.57, SD = 1.81, t = 3.40, df = 135, p = 0.00, d = 0.50); The differences of the emotional state between the two groups were not significant(M an = 6.09, SD = 1.07; M non = 5.79, SD = 1.41, t = 1.64, df = 174, p > 0.05, d = 0.24), indicating that the manipulation of anthropomorphism was successful.

*Social presence.* Through regression analysis, we found that anthropomorphic communication positively contributes to the sense of social presence (β = 0.90, p = 0.00), by the independent sample T-test we further found that the anthropomorphic communication group produced a higher sense of social presence than the non-anthropomorphic group significantly. A significant difference of the social presence emerged between the anthropomorphic and non-anthropomorphic groups (Man = 6.27, SD = 0.94; Mnon = 5.58, SD = 1.66, t = 2.49, df = 146, p = 0.01, d = 0.36), indicating that anthropomorphic communication led to a higher level of social presence.

*Country-of-origin stereotypes.* The developed country group made higher level of evaluations for the target product than the developing country group when the communication condition was non-anthropomorphic(M _non, fr_ = 6.58, SD = 0.67; M_non, vi_ = 5.91, SD = 1.32, t = 2.52, df = 44, p = 0.02, d = 0.65), there are COO stereotypes between the two groups; However, when the communication condition was anthropomorphic, there was no significant difference between the developed country group and the developing countries group(M_an, fr_ = 6.24, SD = 0.90; M_an,vi_ = 6.20, SD = 0.89, t = 0.18, df = 59, p = 0.86 > 0.05, d = 0.04). Anthropomorphic communication attenuates the COO stereotypes and it further validates Hypothesis 1.

**Figure 3:**
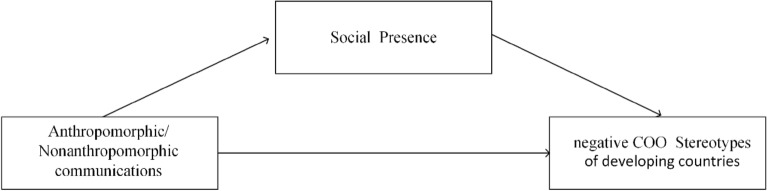
The mediating role of social presence in developing country group.

*The mediating role of social presence.* We used PROCESS Model 4 with 5000 bootstrapping resamples to analyze the mediating effect of social presence^46^, the experiment results showed that:

*In developing country group.* Precious studies have found that social presence made positive impacts on individuals’ trusting beliefs which in turn result in purchase intentions and behaviors^47^. As described in the previous findings, our experimental data confirmed that anthropomorphism excited a higher sense of social presence than non-anthropomorphism, the high sense of social presence generated by anthropomorphic communication could further improve product evaluations in developing country group (β = 0.86, p < 0.05), it improved products evaluations and attenuated the negative stereotypes of developing countries. The sense of social presence mediated the relationship between anthropomorphic/non-anthropomorphic communication and negative COO stereotypes of the developing country group, the mediating effect was significant (95% Confidence CI = 0.25–0.72) and not contained 0. The mediating role of social presence was shown in Fig. 3:

*In the developed country group.* The mediating effect of social presence was not obvious, (95% Confidence CI = -0.11 to 0.60) contains 0. That is because the high sense of social presence can improve product evaluations (β = 0.67, p < 0.05). In the condition of a high sense of  social presence, the communication modality(anthropomorphic or non-anthropomorphic) had an inconspicuous effect on COO stereotypes(p = 0.85 > 0.05), that means the positive COO stereotypes of the developed countries will not be reduced due to the adoption of anthropomorphic communication strategies.

### Study 3

Study 3 is to verify the effect of individuals’ mindset on the relationship between communication modality and COO stereotypes (H3). We took 2 (global mindset vs. local mindset) × 2 (anthropomorphism vs. non-anthropomorphism) × 2 (developed country vs. developing country) between-subjects design. Respondents didn’t guess the purpose of the experiment. We collected 293 valid subjects (age range = 18–69, 59.4% female) for subsequent analysis, each person was paid 7 yuan as a reward. As the previous two studies, information was released by the spokesman via social media, and participants were told that the activity was for promoting a new brand of milk.

#### Stimulating individuals’ local and global mindset

Based on Lamberton’s research, we used benefit-based and attribute-based assortment to stimulate individuals’ local and global mindset^48^. Two groups (benefits group vs. attribute group) of nutritional bars were placed in front of the participants. The participants in different groups were asked to choose three favorite nutritional bars in turn.

*In the attribute-based condition.* A description was provided as follows: “Imagine that you are choosing a nutritional bar in the supermarket, these bars are made by Company B. Do you know what's in the nutrition bar? Nutrition bars are usually described in terms of attributes, when you make a choice, you can pay attention to these attributes of bars, you should read the attribute descriptions of bars carefully before buying.” The researchers divided the nine bars into three categories based on their attributes: chocolate bars, fruit bars, and nut bars. Each type of bars had a description following their names. For example, the description of fruit bars indicated: "Many bars contain a wide variety of fruits, such as the common pear, strawberry, grapefruit, or exotic agave."

*In the benefit-based condition.* A description was provided as follows: “Imagine that you are choosing a nutritional bar in the supermarket, these bars are made by Company B. Do you know what can nutrition bars do? In general, nutrition bars are defined and classified according to their functions, and we should pay attention to their functions when choosing nutrition bars. Here are three types of nutrition bars with their functions.” The participants were shown muscle-boosting bars, energy-boosting bars, and fat-burning bars in the same way. Each type of bars had a description following their names. For example, the description of fat-burning bars was: “The bars can speed up consumer's metabolism, so the body can burn calories more efficiently.”

The researchers let participants choose their favorite bars with no time limit until they picked the three ones they liked best. After identifying their favorite bars in turn, the researcher asked the participants a question to make sure that they confirmed their choice. In the attribute-based group, the checking item was: “Before I start shopping, I know which attribute I would like to choose in advance, for example, I’d like a fruit bar, a nut bar, or a candy bar”. In the benefit-based group, the checking question was: “Before I start shopping, I know what benefits I want to gain from the nutrition bars in advance, for example, I want to burn fat, build muscle, or boost energy.” Answer “0” is strongly disagree and “1” is strongly agree.

After determining the bars they preferred, participants completed a short Behavior Recognition Scale (BIF), which measured their level of construal mindset^49^. We selected ten questions from the Behavior Recognition Scale, participants were presented with the information as follows: “Choose the answer that comes to your mind first”, and then they filled in the answer list. Each question had two choices, the benefit-oriented answer and the attribute-oriented answer. The choice chosen by the participant was assigned 1 point, and the other pointed 0.

"Erick" was selected as the virtual brand, and it was introduced by the way of anthropomorphic or non-anthropomorphic communication. In the group of anthropomorphic condition, participants were displayed a cartoon milkman who was clapping with a cartoon biscuit man, and the spokesman via social media said: “Hi, I’m Erick, take me with you, let’s enjoy the vitality and happiness together.” For the non-anthropomorphic condition, the photo showed a box of milk and a biscuit, and the spokesman said: “The brand of the milk is Erick, have it, and you will feel happy and energetic.” We chose Canada and Mexico to represent the developed country information and the developing country information respectively.

Participants were arranged randomly into one of eight groups: local, anthropomorphic, Mexico (N_lnm_ = 34); local, anthropomorphic, Canada (N_lnc_ = 37); local, non-anthropomorphic, Mexico (Nl_nm_ = 39); local, non-anthropomorphic, Canada (N_lnc_ = 39); global, anthropomorphic, Mexico (N_lnm_ = 35); global, anthropomorphic, Canada (N_lnc_ = 38); global, non-anthropomorphic, Mexico(N_lnm_ = 34); global non-anthropomorphic, Canada (N_lnc_ = 37). We tested the evaluation of the product in different groups, the experimental procedure was consistent with previous two studies, the only difference of Study 3 lies in the priming stimulus of psychological mindset at the beginning of the investigation.

#### Results and discussion

##### Individuals' mindset

*Global mindset.* For global mindset stimulation, scores of global-mindset-oriented (benefit-oriented) items were significantly higher than local-mindset-oriented (attribute-oriented) items (M_global_ = 5.33, SD = 2.69; M_local_ = 4.59, SD = 2.51, t = 2.42, df = 291, p = 0.02, d = 0.28). That meant participants’ global mindset was provoked successfully by benefit-oriented items;

*Local mindset.* For local mindset stimulation, scores of global-mindset-oriented (benefit-oriented) items were significantly lower than local-mindset-oriented (attribute-oriented) items (M_global_ = 4.64, SD = 2.71; M_local_ = 5.40, SD = 2.53, t = -2.50, df = 291, p = 0.01, d = -0.29). That meant the participants’ local mindset was provoked successfully by attribute-oriented stimulation.

The results are consistent with scholar Liu’s previous study that benefit-oriented stimulation leads to individuals’ global mindset, and attribute-oriented stimulation leads to individuals’ local mindset^39^.

*Anthropomorphism.* We conducted an independent sample T-test on the level of anthropomorphic communication. Individuals in the anthropomorphic group reported a higher level of anthropomorphic tendency than those in the non-anthropomorphic group (M_an_ = 5.08, SD = 1.16; M_non_ = 4.11, SD = 1.71, t = 5.66, df = 261, p = 0.00, d = 0.66). The degree of emotional state between the two groups was not different obviously (M_an_ = 5.27, SD = 0.90; M_non_ = 5.07, SD = 0.97, t = 1.80, df = 291, p > 0.05, d = 0.21), that verified the success of the anthropomorphic manipulation.

*Country-of-origin stereotypes and the moderating role of mindset.* We conducted 2 (anthropomorphic vs. non-anthropomorphic) × 2(local mindset vs. global mindset) × 2(developed country vs. developing country) multivariate analysis of variance with the product evaluation of the origin country as the dependent variable. The results showed a significant interactive effect between communication modality and individuals’ mindset (F = 2.79, df = 1, p = 0.096 < 0.1). We conducted further simple effect analysis, results were shown in Table 2.

**Table 2:** Simple effect analysis of communication modality and mindset.

Global/local	Anthropomorphic/non-anthropomorphic	Country information		AD	Standard errors	p
Global	Anthropomorphic	Developed country	Developing country	0.245	0.227	0.282
		Developing country	Developed country	− 0.245	0.227	0.282
	Non-anthropomorphic	Developed country	Developing country	0.401	0.230	0.083
		Developing country	Developed country	− 0.401	0.230	0.083
Local	Anthropomorphic	Developed country	Developing country	0.045	0.230	0.845
		Developing country	Developed country	− 0.045	0.230	0.845
	Non-anthropomorphic	Developed country	Developing country	− 0.090	0.220	0.683
		Developing country	Developed country	0.090	0.220	0.683

**Figure 4:**
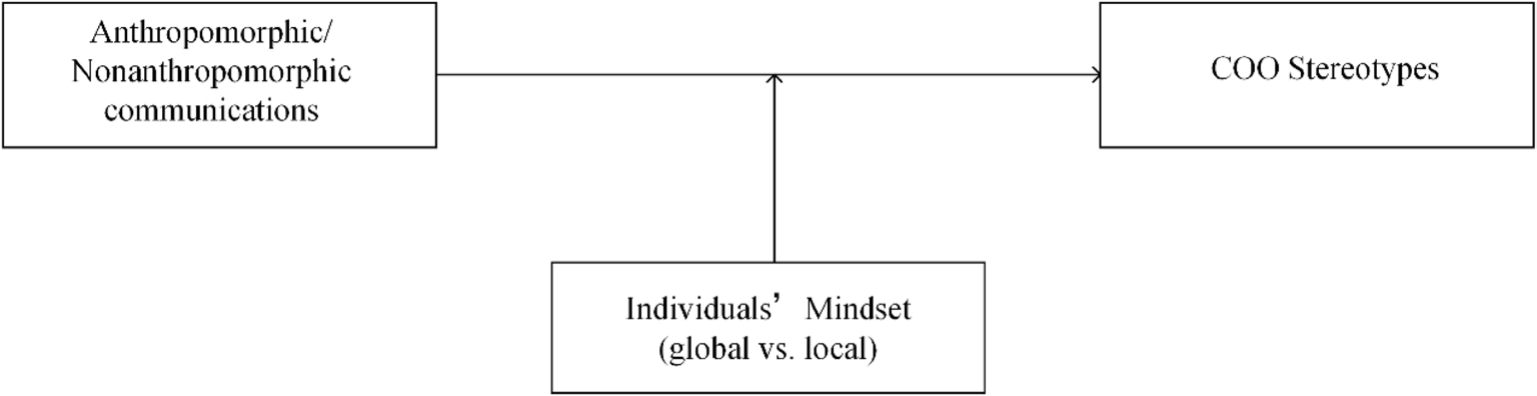
The moderating role of individuals’ mindset.

From the results, we conclude that: **when individuals’ global mindset is elicited**, in the non-anthropomorphic group, the product evaluation in the developed country condition and developing country condition is different significantly (p = 0.083 < 0.1), the product evaluation in the developed country condition is higher than that in the developing country condition. However, in the anthropomorphic communication group, the difference between the two conditions is not obvious. That means when individuals’ global mindset is elicited, anthropomorphic communication has a significant impact on country-of-origin effects; **when individuals’ local mindset is elicited**, the product evaluation in the developed and developing country condition is not different significantly, whether in anthropomorphic communication group (p = 0.854 > 0.1) or non-anthropomorphic group (p = 0.683 > 0.5). That means when individuals’ local mindset is elicited, the effect of anthropomorphic communication on COO stereotypes is no longer obvious. The moderating effect of individuals’ mindset is shown in Fig. 4:

Study 3 verified the moderating effect of individuals’ mindset (local vs. global) on the relationship between communication modality (anthropomorphism vs. non-anthropomorphism) and COO stereotypes. The effect of anthropomorphic communication on COO stereotypes is obvious only when individuals’ mindset is global.

## Conclusion and discussion

### Conclusion

Study 1 confirms that anthropomorphic communication influences COO stereotypes. It weakens COO stereotypes not only for developing countries but also for developed countries. Study 2 explains the internal theoretical mechanism of anthropomorphic communication, social presence, and COO stereotypes. It verifies that anthropomorphic communication could trigger a sense of social presence, and the high sense of social presence reduces the negative stereotypes of developing countries; Study 3 confirms the effect of individuals’ mindset on moderating the relationship between communication modality and COO stereotypes. Anthropomorphic communication plays a significant role on COO stereotypes only when individuals with a global mindset.

### Theoretical implications

1. It enriches the connotation of schema theory and the application scenarios of anthropomorphic communication. By researching the schema theory, we construct the application mechanism of the anthropomorphic strategy on COO stereotypes, find out factors affecting the effectiveness of anthropomorphic communication strategy on COO stereotypes, and propose theoretical suggestions for adopting anthropomorphic strategies accurately. In this paper, schema theory as a theoretical support and main theoretical clue, connects the two variables (anthropomorphism and stereotypes) from different research field, and find a new way to investigate influencing factors on COO stereotypes, the connotation of schema theory is further enriched; Until now, researches on the effectiveness of anthropomorphism on influencing COO stereotypes are still limited. This study further enriches the research findings in the anthropomorphic field and puts forward feasible theoretical suggestions combining COO stereotypes with psychological elements. The application scenarios of anthropomorphism are further enriched.
2. Based on studying the influence of social presence stimulated by emotional interaction, the construct of social presence is introduced to the COO field, which extends the theoretical branch of emotional interaction in psychology. Previous studies about social presence were mainly on individuals' online loyalty^50^, behavioral intention, or actual brand experiences^3,51^, etc. In this research, we studied the role of social presence induced by anthropomorphic communication and found its mediating role between anthropomorphism and negative COO stereotypes. The impact of psychological variation resulting from interaction effects on attenuating negative COO stereotypes is described, it could broaden the psychological branch of emotional interaction.
3. Based on studying the role of individuals’ mindset, the paper provides new insights to investigate psychological factors for the relationship between anthropomorphism and COO stereotypes. We introduce individuals’ mindset (local/global) to the research field of anthropomorphic communication, focusing its moderating role on regulating the relationship between anthropomorphism and COO stereotypes, and put forward reasonable theoretical suggestions for brand advertising.

### Practical implications

1. It gives implications for enterprises and advertisers to use anthropomorphic strategies accurately and cautiously. Experiment results show that anthropomorphic communication via social media reduces COO stereotypes for both developing countries and developed countries. In the global marketing, enterprises in developing countries can adopt anthropomorphic strategies to reduce product stereotypes while developed country enterprises need to be more cautious to do this. At the same time, advertising content, interactive communication, and individuals’ psychological factors such as perceived social presence and mindset need to be considered comprehensively.
2. It provides suggestions for advertisers to consider the mediating role of social presence induced by anthropomorphic communications. In developing countries, the sense of social presence mediates the relationship between anthropomorphic communication and COO stereotypes, the sense of social presence triggered by interactive communication could reduce negative COO stereotypes of developing countries, and enterprises could improve international competitiveness by adopting anthropomorphic communication. For developed countries, the sense of social presence improves product evaluations and counteracts the weakening effect of anthropomorphism on positive COO stereotypes by conducting interactive activities.
3. It gives implications of considering psychological factors that influence the validity of anthropomorphism on COO stereotypes for international marketing. Different advertising content could stimulate different individuals’ mindset. When the global mindset (emphasize products’ benefit characteristics) of individuals is excited by advertisements, adopting anthropomorphic communication strategies could be suitable for developing countries; when individuals’ local mindset is excited by advertisements (emphasize products’ attribute characteristics), the difference of using anthropomorphic strategies and non-anthropomorphic strategies is not obvious.

### Limitations and future research

This research reveals the influence of anthropomorphic communication on COO stereotypes. We merely discuss the moderating effect of individuals’ mindset and the mediating role of perceived social presence, other factors, such as personal knowledge, personal temperament, and different types of products could be explored in depth.

## Data Availability

The datasets generated and/or analyzed during the study are available from the corresponding author upon reasonable request.
